# Editorial Notes

**Published:** 1925

**Authors:** 


### Editorial Notes.

**The "Bristol Medico-Chirurgical Journal" for 1926** and onwards will have a somewhat larger page, and will be printed in a larger type on a faced paper. We feel sure that our readers will appreciate the better presentation of our subject-matter, together with a more pleasing cover. The Editorial Committee have had these improvements under consideration for some time, but hitherto the difficulties involved in publication and the increased cost of labour and paper have delayed their adopting these much-desired alterations and improvements; while throughout the Great War it was only possible to continue publication at all by the exercise of rigid economy.

**Bristol Medical School Annual Dinner.** On December 3rd Professor E. W. Hey Groves presided at a large gathering of past and present students, well supported by the members of the clinical teaching staff. The guest of the evening was Sir Berkeley Moynihan, Bart., K.C.M.G., on whom last summer an Honorary Degree was conferred by the Council and Senate of the University of Bristol. After the Royal Toast the Chairman proposed the toast of the guest of the evening.

He said that he was a proud and happy man to have been chosen as President and to have the privilege of proposing the health of one who for many years had been his ideal as a surgeon, teacher and orator. He asked that they should drink his health because of the great and wonderful influence Sir

Berkeley had upon surgical thought, teaching and practice. The President said that Sir Berkeley was responsible for founding three great movements: the Surgical Club, the *British Journal of Surgery* (published and edited in their city) and the Association of Surgeons.

Sir Berkeley's influence during the war was referred to as being of a unique character ; as a provincial surgeon he was chosen by the War Office to be Chairman of the Consultants' Council, and did wonderful work, in association with Sir Robert Jones, in the organisation of the treatment of orthopædic surgery in the military world.

Speaking of art and religion, the President said that they had this in common. Art was an attempt to realise the ideal ; religion was the worship of the ideal ; and in this sense Sir Berkeley was the most religious man he knew. The President spoke with pride of the conferment of the honorary degree of Doctor of Laws of Bristol University on Sir Berkeley, and said that already his advice had been sought in the matter of the clinical teaching of medicine and surgery. Concluding his remarks with a humorous reference to his unique position as a friend of Sir Berkeley's for fifteen years, yet still retaining his full proportion of internal organs (being careful to add that this was not said in any boasting spirit), the President called upon the company to drink to the health of their guest.

Sir Berkeley Moynihan, who was greeted most warmly on rising to respond, remarked that Mr. Hey Groves was the first of many people in all parts of the world who had ever made a speech about him that he felt was appropriate to the occasion, and he considered it so for several reasons. He realised that what a man did for himself and by himself mattered very little indeed. Books and articles, by the time they were in print, said Sir Berkeley, never seemed to represent one's self worthily, but rather made one feel great dissatisfaction with all one's literary efforts. It was still worse with the spoken word, he said, which might seem warm when uttered, but cold on the morrow if remembered at all.

What really counted were the things a man left behind him in the heart, in the mind and in the work of others. He told how, in his early days, he had set himself a certain ideal.

Ideals were not for attainment but for pursuit, and he had chased an ideal in his surgical life, which was that he should leave behind him in his own hospital men better than himself to carry on the work after he had done.

Sir Berkeley spoke in high praise of Mr. Hey Groves's achievements, particularly in connection with the *British Journal of Surgery,* which he had been told, in countries other than his own, was the best surgical journal to-day. He referred to the publishers (Messrs. J. Wright & Sons), whose centenary was being celebrated this year, and remarked that he would like to thank Mr. Wright for his share in the *British Journal of Surgery.* As an honorary graduate of the University of Bristol, Sir Berkeley said he was anxious to meet his confrères, and glad of this opportunity of doing so.

Sir Berkeley also paid high tribute to Mr. Greig Smith, of the Bristol School, as the greatest surgeon of his day. Speaking of the school curriculum Sir Berkeley urged the necessity for postgraduate work, and the great value of a research scholarship (for which he was clamouring in Leeds) in keeping the graduates at least two or three years after qualification, which he considered to be a great need. He concluded his remarks with greetings from Leeds, and drank to the health of the Bristol Medical School.

Mr. W. S. V. Stock, replying to the toast, expressed in a few well-chosen words the thanks of the Bristol Medical School and their appreciation of Sir Berkeley's acceptance of the invitation to be the Guest of the Evening. He spoke with pride of the early days of the Bristol Medical School, the foundation of which was, in fact, the foundation of the Bristol University of to-day.

Mr. Jacomb jointly responded.

Mr. J. Drew Smythe proposed the health of the President.

Mr. Hey Groves suitably replied, and thereafter enjoined the company to proceed to the Spa Hotel. Here the wives of the staff of the Faculty of Medicine held a reception and dance, a most enjoyable ending to a highly successful evening.

